# Efficient Amplification in Soai's Asymmetric Autocatalysis by a Transient Stereodynamic Catalyst

**DOI:** 10.3389/fchem.2020.615800

**Published:** 2020-12-09

**Authors:** Oliver Trapp

**Affiliations:** ^1^Department of Chemistry, Ludwig-Maximilians-University Munich, Munich, Germany; ^2^Max-Planck-Institute for Astronomy, Heidelberg, Germany

**Keywords:** asymmetric autocatalysis, non-linear effects, kinetics, organometallic chemistry, Soai's reaction

## Abstract

Mechanisms leading to a molecular evolution and the formation of homochirality in nature are interconnected and a key to the underlying principles that led to the emergence of life. So far proposed mechanisms leading to a non-linear reaction behavior are based mainly on the formation of homochiral and heterochiral dimers. Since homochiral and heterochiral dimers are diastereomers of each other, the minor enantiomer is shifted out of equilibrium with the major enantiomer by dimer formation and thus a reaction or catalysis can be dominated by the remaining molecules of the major enantiomer. In this article a mechanism is shown that leads to homochirality by the formation of a highly catalytically active transient intermediate in a stereodynamically controlled reaction. This is demonstrated by Soai's asymmetric autocatalysis, in which aldehydes are transformed into the corresponding alcohols by addition of dialkylzinc reagents. The mechanism of chirogenesis proposed here shows that an apparently inefficient reaction is the best prerequisite for a selection mechanism. In addition, stereodynamic control offers the advantage that the minor diastereomeric intermediate can be interconverted into the major diastereomer and thus be stereoeconomically efficient. This is supported by computer simulation of reaction kinetics.

## Introduction

**Figure 1:**
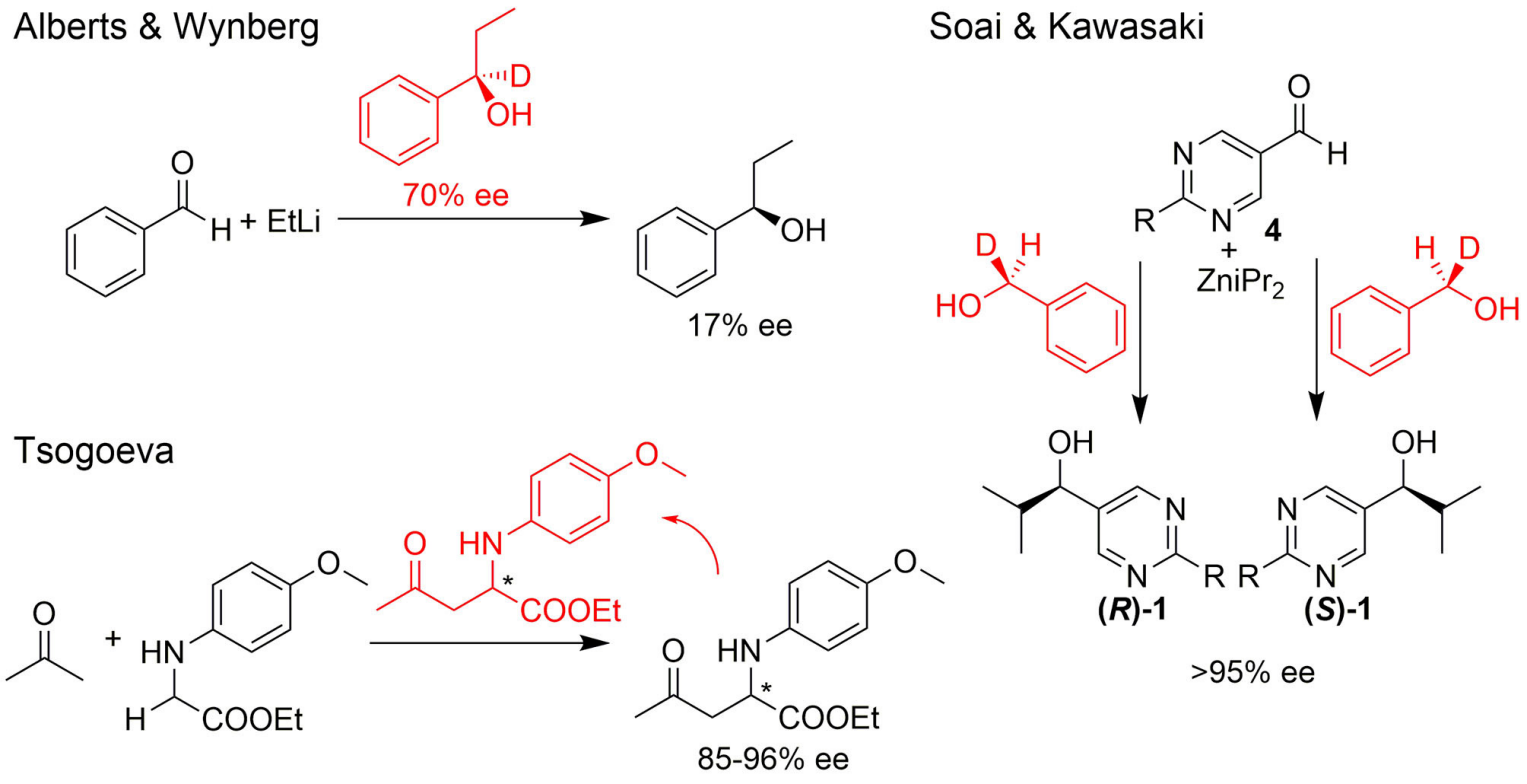
Selected examples for asymmetric amplification of initial small imbalances by autocatalytic reactions. R = –C=C–C(CH_3_)_3_.

The single-handedness of molecular building blocks, such as amino acids and sugars, in biologically relevant metabolisms and (polymeric) structures is considered the signature of life and an important prerequisite for the emergence of life (Hegstrom, 1984; Blackmond, 2004, 2011; Kawasaki et al., 2008; Hawbaker and Blackmond, 2019; Karunakaran et al., 2019; Teichert et al., 2019). In the absence of chiral directing forces, an abiotic process provides a racemic mixture. Therefore, one of the most exciting questions is how biological homochirality developed from a predominantly achiral environment. Several theoretical approaches to this question have been investigated experimentally over the last decades and many findings show how enantiomer enrichment may have occurred by physical processes or chemical reactions. Some of the most promising theoretical proposals for asymmetric amplification of initial small imbalances are autocatalytic reactions (cf. Figure 1) (Alberts and Wynberg, 1989; Soai and Kawasaki, 2008; Tsogoeva, 2010; Bissette and Fletcher, 2013). A comprehensive review of such processes was recently compiled by Blackmond (2020).

**Figure 2:**
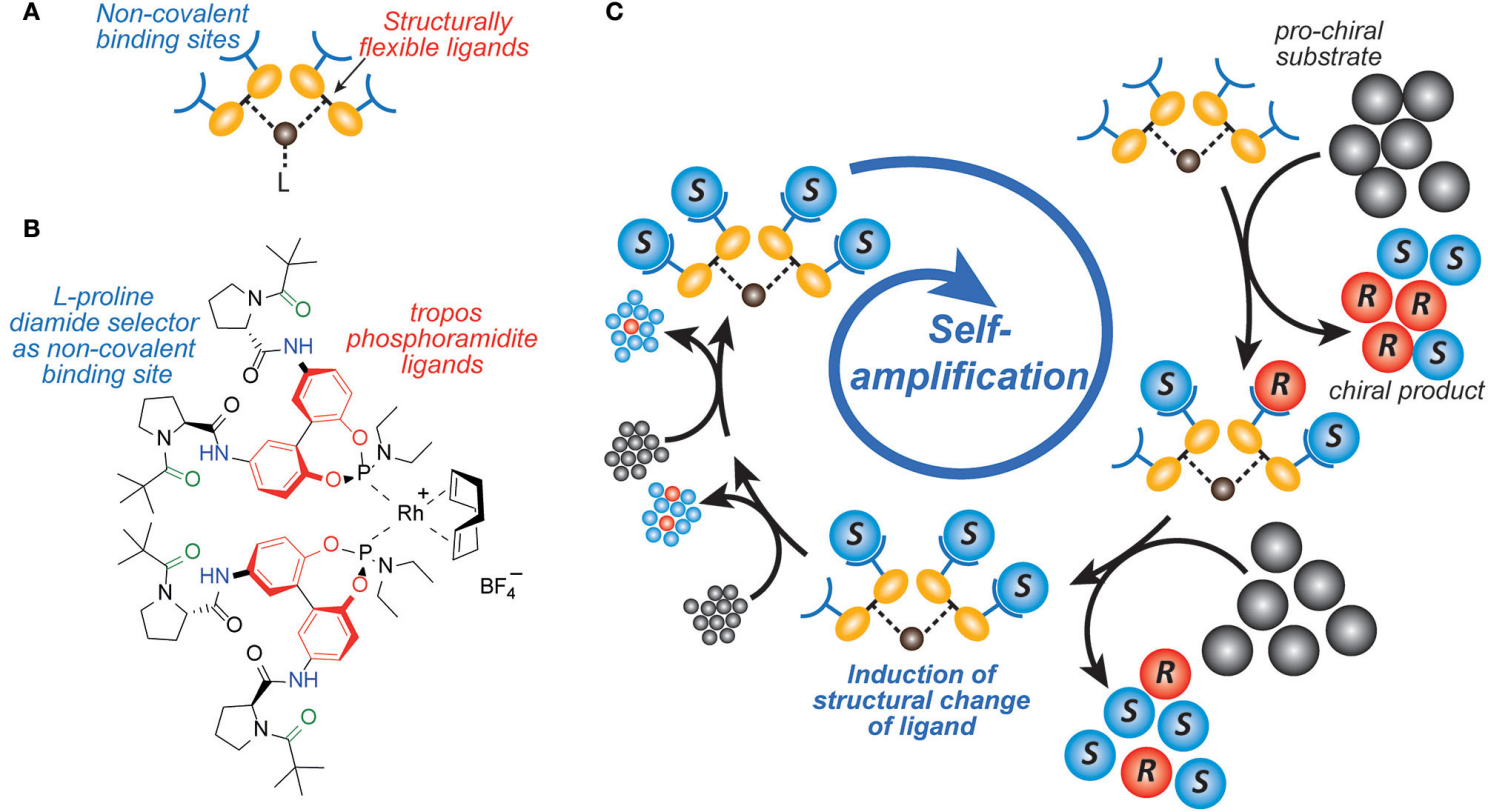
Enantioselective self-amplifying catalysis. **(A)** Design of a stereodynamic self-amplifying catalyst with non-covalent binding sites to interact with the product enantiomers formed in the catalyzed reaction. **(B)** Structure of a rhodium(I) complex with tropos Phosphoramidite ligands which are self-aligned upon preferential interaction of the non-covalent binding sites with one of the formed product enantiomers. **(C)** Steps leading to enantioselective self-amplifying catalysis. After activation of the pre-catalyst the product is formed in no or low enantioselectivity. The catalyst interacts at the product interaction sites with the initially formed product. This product-catalyst interaction induces a change in the catalyst structure, which leads to enhanced enantioselectivity of the catalyst.

Early mechanistic concepts of such reactions with positive nonlinear effects (Blackmond, 2010) were discussed by Noyori (Kitamura et al., 1989; Mikami et al., 2000) and Kagan (Girard and Kagan, 1998; Satyanarayana et al., 2009). Predominantly the formation of reversible monomer-dimer association complexes was considered. Frank postulated a theoretical model (Frank, 1953) that leads to a spontaneous asymmetric synthesis by forming dimers from their monomer building blocks, for example by intermolecular interaction. If these monomers have the same configuration, the dimers are homochiral, or if the monomers have opposite configurations, heterochiral dimers are obtained. Since these dimers are diastereoisomeric to each other, they have different intrinsic properties, which are reflected for example in their formation and decomposition rates, their solubilities, their chiroptic properties. Thus, the formation of a heterochiral dimer from an enantiomerically enriched mixture can increase the enantiomeric excess of the free monomers. Ideally, this process could even lead to the result that only the main enantiomer remains monomeric in a solution and the heterochiral dimer precipitates as insoluble solid. If the remaining major enantiomer is catalytically active, this process can lead to the starting point of a highly efficient amplification. However, this would be an exceptionally rare case. In recent years, we have developed catalysts decorated with chiral recognition units to recognize and transfer the chirality of the reaction product generated in the catalysis to the stereodynamic unit of the catalyst (Maier and Trapp, 2014; Storch and Trapp, 2015, 2017, 2018; Storch et al., 2015, 2016a,b; Scholtes and Trapp, 2019a,b,c,d). This induces a shift in the equilibrium of the stereodynamic catalyst by the recognized chirality (cf. Figure 2). In this way, it was possible to develop self-amplifying catalytic systems which aligned their configuration dynamically during catalysis and thus formed a preferred enantiomer from the prochiral substrate (Scholtes and Trapp, 2020). In the course of this research we have been working intensively on the elucidation of the mechanism of the Soai reaction (Trapp et al., 2020). Saoi's asymmetric autocatalysis (Soai et al., 1995; Shibata et al., 1997) is a highly unusual reaction. In this reaction pyrimidine-5-carbaldehydes **4** are reacted with diisopropyl zinc in the presence of catalytic amounts of the corresponding pyrimidine alcohol **1** with low *ee*. Asymmetric autocatalytic amplification of the enriched enantiomer yields the pyrimidine alcohol **1** with enhanced *ee*. Several mechanistic models were proposed to explain the extraordinary behavior of this reaction (Blackmond et al., 2001; Blackmond, 2006; Ercolani and Schiaffino, 2011; Gehring et al., 2012; Micheau et al., 2012; Gridnev and Vorobiev, 2015; Athavale et al., 2020).

**Figure 3:**
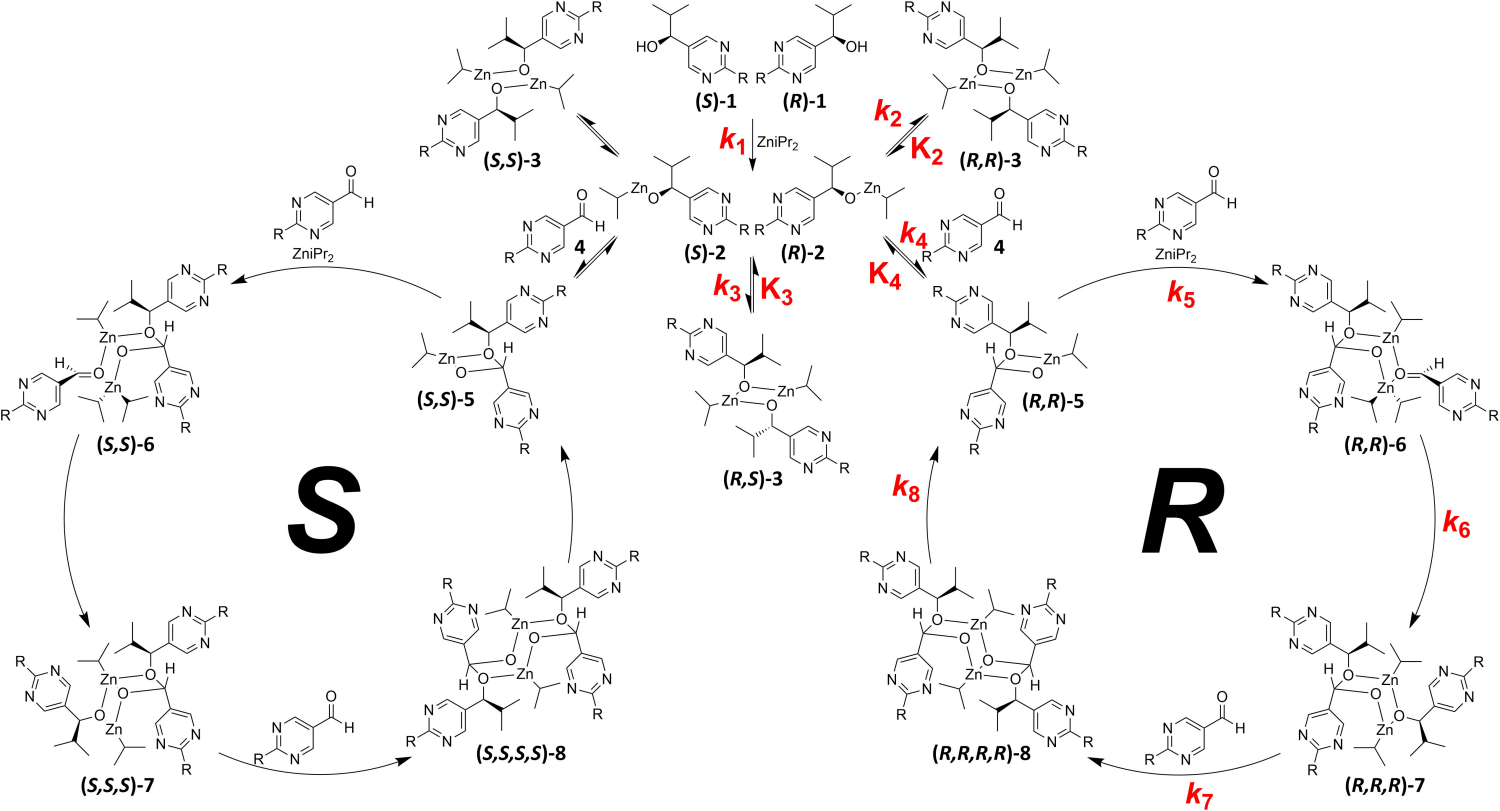
Proposed mechanism of the Soai-reaction with the formation of the transient Zn-hemiacetalate catalyst **5**. Establishing of the equilibrium of homochiral **(*R***, ***R*)-3**/ **(*S***, ***S*)-3** and heterochiral **(*R***, ***S*)-3** dimers of the isopropylzinc pyrimidyl alkoxides **2** and establishing the autocatalytic cycle after formation of the hemiacetalate **5**. R = –C=C–C(CH_3_)_3_.

By comprehensive kinetic and mass spectrometry experiments, we recently identified and monitored the formation of a transient catalyst that forms a hemiacetalate-zinc complex **5** by reaction of pyrimidine-5-carbaldehyde **4** with the corresponding pyrimidine alcoholate **2** (Trapp et al., 2020). The dynamic behavior of the formed hemiacetals **5** is of particular interest and was investigated by dynamic HPLC (DHPLC). The mass spectrometric detection of the substrates and the product as well as the intermediates occurring during the reaction enabled the derivation of a reaction mechanism (cf. Figure 3). First of all, the alcoholate **2** is formed from the alcohol added as an additive to diisopropyl zinc, which can form homochiral **(*R***, ***R*)-3**/**(*S***, ***S*)-3** or heterochiral dimers **(*R***, ***S*)-3**. The zinc alcoholate **2** reacts slowly with the aldehyde **4** in an equilibrium reaction to form the hemiacetalate **5**, which first adds another molecule of diisopropyl zinc and aldehyde **4** to form complex **6** and is then enantioselectively alkylated to complex **7**. In a further step, another molecule of aldehyde is added and forms a dimeric hemiacetalate complex **8** that decomposes into its monomeric hemiacetalates **5**, establishing an autocatalytic cycle. The reaction product, the alcoholate **2** and its dimers **3**, is continuously formed by the back reaction of the hemiacetalate **5**/alcoholate-aldehyde (**2**–**4**) equilibrium.

This contribution will focus on the dynamics of hemiacetal formation and will investigate the influence of reaction kinetics and equilibrium on the autocatalytic reaction and the enantiomeric excess *ee*.

## Materials and Methods

### General

Reagents and solvents were obtained from Sigma-Aldrich (Taufkirchen, Germany), ABCR (Karlsruhe, Germany), and Alfa Aesar (Karlsruhe, Germany) and were used without further purification. Standard Schlenk techniques were used for air sensitive reactants. Glass ware was heated prior to use and the syntheses were carried out under an argon atmosphere.

NMR spectra were recorded on Bruker Avance 600 and 500 MHz spectrometers.

HPLC and HPLC-MS measurements were performed on an Agilent 1200 Infinity HPLC equipped with a binary pump, an autosampler (Agilent HiP+), a thermostatted column oven and a photodiode array detector (DAD). All operations were controlled by the Agilent Chemstation software. Enantioselective separations were performed on an immobilized chiral stationary phase (CSP) cellulose tris(3,5-dichlorophenylcarbamate), Chiralpak IC-3 (15 cm, I.D. 4.6 mm, particle size 3 μm, Chiral Technologies, Parc d'Innovation, Bd Gonthier d' Andernach, 67400 Illkirch Cedex, France) using *n*-hexane/2-propanol 60:40 (v/v) as mobile phase at a flow rate of 1.0 mL·min^−1^.

### Evaluation of the Dynamic HPLC Profiles

Dynamic HPLC traces were analyzed by the unified equation, which allows the direct calculation of reaction rate constants *k*_1_ and *k*_−1_ and Gibbs activation energies Δ*G*^+^ for all types of (pseudo) first-order reactions taking place in chromatographic systems, regardless of the initial concentrations of the interconverting analytes A and B and the equilibrium constant *K*_A/B_. A detailed description of the derivation is given in literature (Trapp, 2006a,b,c,d; Trapp et al., 2009).

## Results and Discussion

**Figure 4:**
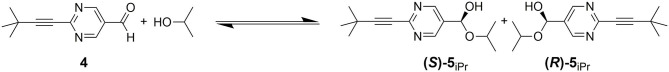
Hemiacetal formation of 2-(*tert*-butylacetylene-1-yl)pyrimidyl-5-carbaldehyde 4 with 2-propanol.

During the HPLC separation of pyrimidine-5-carbaldehyde **4** with 2-propanol in the mobile phase a second peak is observed which is connected to the peak of pyrimidine-5-carbaldehyde **4** by a plateau formation. If this separation is performed using a chiral stationary phase, e.g., with Chiralpak IC-3, then the separation of the newly formed peak into two peaks is observed, which indicates, that enantiomers have been formed (cf. Figure 4). Using HPLC-MS, the newly formed peaks can be clearly assigned to the hemiacetals **(*R*)-5**_iPr_ or **(*S*)-5**_iPr_ formed from the 2-propanol of the mobile phase and the pyrimidine-5-carbaldehyde **4**. The observation of hemiacetals is important because chromatographic techniques can be used to study and screen the reactivity of the formation of hemiacetals from aldehydes and alcohols. It can be expected that the formation of hemiacetalates **5** from aldehydes **4** and zinc alcoholates **2** occurs with similar reactivity due to the electronic properties of the aldehydes **4** and therefore the ability of formation can be directly correlated.

**Figure 5:**
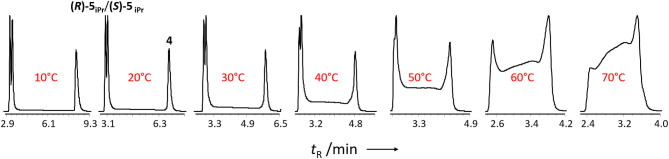
Temperature-dependent enantioselective DHPLC measurements of the formation of the hemiacetal starting from 2-(*tert*-butylacetylene-1-yl)pyrimidyl-5-carbaldehyde 4 with 2-propanol. Experimental conditions: Chiralpak IC-3 (15 cm, I.D. 4.6 mm, particle size 3 μm), *n*-hexane/2-propanol 60:40 (v/v), flow 1.0 mL·min^−1^.

To determine the reaction rate of the hemiacetal formation and decomposition of the aldehyde **4** in presence of 2-propanol, we performed temperature-dependent enantioselective dynamic HPLC (DHPLC) (Trapp et al., 2001; D'Acquarica et al., 2006; Wolf, 2008; Trapp, 2013) measurements. As can be seen from the elution profiles (cf. Figure 5) a pronounced plateau formation with increasing reaction temperature can be observed.

**Figure 6:**
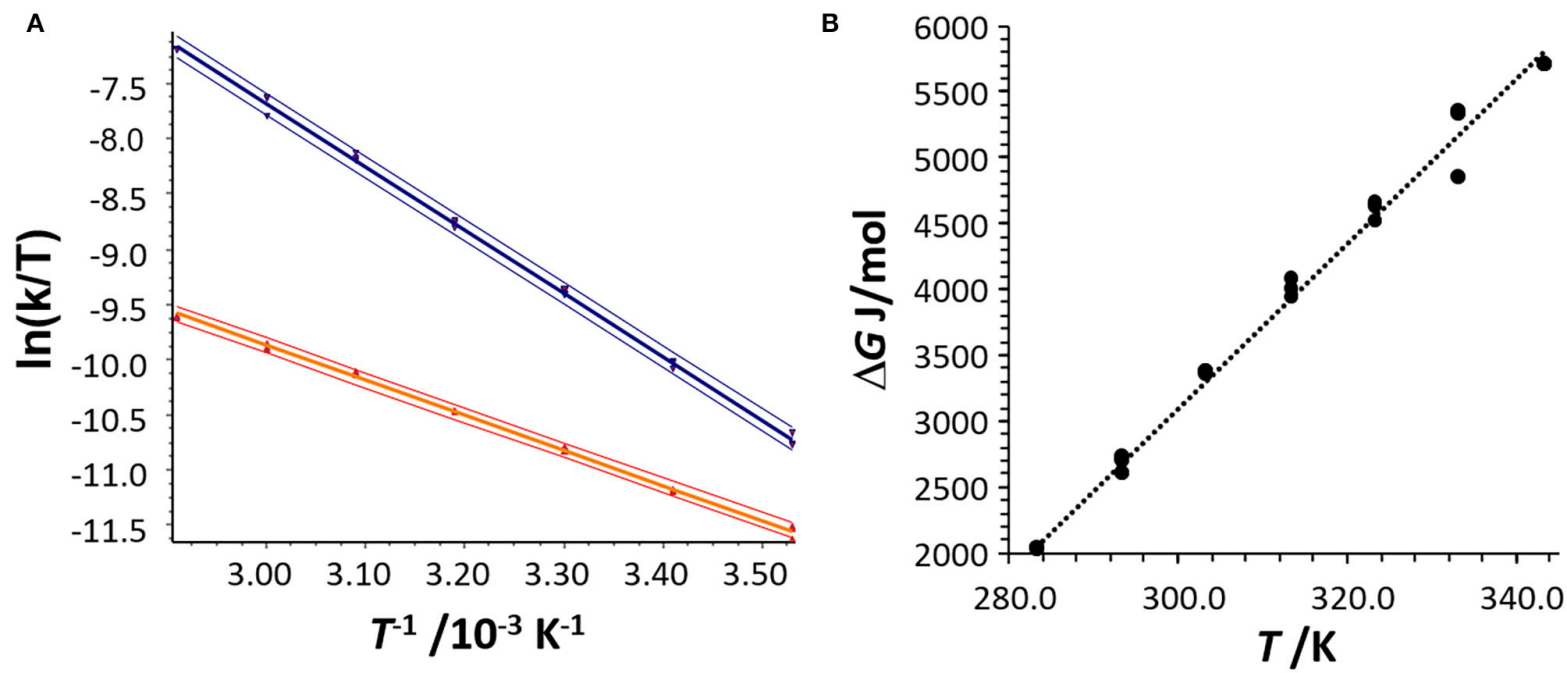
**(A)** Eyring plot for the determination of the activation parameters Δ*H*^+^ and Δ*S*^+^ of the hemiacetal formation (red data points) and the hemiacetal decomposition (blue data points) obtained from the DHPLC experiment considering the concentration of 2-propanol. The upper and lower curves represent the error bands of the linear regression with a level of confidence of 95%. For the linear regression 21 data points were considered. **(B)** Determination of the thermodynamic parameters Δ*H* and Δ*S* by plotting the Gibbs free energy Δ*G* as a function of *T*.

The kinetic analysis was performed by analysis with the unified equation of chromatography considering a pseudo-first-order reaction because of the excess of 2-propanol in the mobile phase. This allowed the determination of the reaction rate constants, e.g., *k*_1_ (293 K) = 4.1 × 10^−3^ (mol × s)^−1^ and *k*_−1_(293 K) = 1.3 × 10^−2^ s^−1^, and the determination of the activation enthalpies Δ*H*^‡^ for the hemiacetal **5**_**i**Pr_ formation and decomposition via the slope and the activation entropies Δ*S*^‡^ via the intercept of the Eyring plots [ln(*k*/*T*) vs. 1/*T*] (cf. Figure 6A). Deviations of the activation parameters Δ*H*^‡^ and Δ*S*^‡^ have been calculated by error band analysis of the linear regression with a level of confidence of 95%. The activation parameters of the hemiacetal **5**_**i**Pr_ formation were determined to be Δ*H*^‡^ = 26.3 ± 0.2 kJ/mol and Δ*S*^‡^ = −195 ± 34 J/(K × mol) (*r* = 0.9990, residual deviation *s*_y_ = 0.0306) and for the backward reaction, the hemiacetal **5**_**i**Pr_decomposition, the activation parameters were determined to be Δ*H*^‡^ = 47.7 ± 0.2 kJ/mol and Δ*S*^‡^ = −112 ± 1 J/(K × mol) (*r* = 0.9994, *s*_y_ = 0.0630). These activation parameters indicate, that the formation of hemiacetal **5**_**i**Pr_ from **4** is an endergonic process, which is highly dynamic. The thermodynamic parameters of the formation of the hemiacetal **5**_**i**Pr_ were determined by linear regression of the thermodynamic Gibbs free energies Δ*G*(*T*), obtained from the equilibrium constants *K*, vs. the temperatures *T* (correlation coefficient *r* = 0.9949) to be Δ*G*^0^ = 3 kJ/ mol, Δ*H*^0^ = −15.6 kJ/mol and Δ*S*^0^ = −62.5 J/(K × mol) (cf. Figure 6B).

It is important to note that this reversible process of hemiacetal formation creates a stereocenter, which leads to the formation of diastereomers in the case of the reaction with a chiral alcohol. It is therefore obvious that a reaction with the corresponding alcohol or alcoholate in the Soai reaction leads to diastereomeric hemiacetals or hemiacetalates **5**. This endergonic process leads to the formation of more hemiacetal, which are well coordinating chiral ligands, at low temperature. This is consistent with the higher reaction rates observed at low temperatures in the Soai reaction.

We have extended the investigation of hemiacetal formation to ^1^H NMR studies of the chemical equilibrium. For this purpose, 5 mg each of benzaldehyde, pyrimidyl-5-carbaldehyde **4**_**H**_ and 2-(*tert*-butylacetylene-1-yl)pyrimidyl-5-carbaldehyde **4** were mixed with 0.5 ml methanol-d_3_. The results of the equilibrium adjustment after 6 h are summarized in Table 1. As can be clearly seen, the hemiacetals are formed with yields of 9% in the case of benzaldehyde and, remarkably, 95% of the corresponding hemiacetals of pyrimidyl-5-carbaldehydes **4**_**H**_ and 2-(tert-butyl acetylene-1-yl)pyrimidyl-5-carbaldehyde **4**, respectively. This reveals the unique properties of pyrimidine-5-carbaldehydes, which are excellent at forming hemiacetals. In a further experiment, 20 mg each of pyrimidyl-5-carbaldehyde **4**_**H**_ and 2-(*tert*-butyl acetylene-1-yl)pyrimidyl-5-carbaldehyde **4** were mixed with 5 eq. 2-methyl-1phenyl propanol in anhydrous toluene-d_8_. Toluene was chosen as solvent to achieve reaction conditions comparable to the Soai reaction. The corresponding diastereomeric hemiacetals are obtained in 9 and 11% yield, respectively. The equilibrium constants *K* of the formation of the hemiacetals described here are summarized in Table 1. It is important to note that we focused here on the analysis of the hemiacetals instead of the hemiacetalates **5**, which can be observed by *in-situ* mass spectrometric investigation of the Soai reaction. In the case of the diastereomeric hemiacetals **5**, the formation of a major and a minor diastereomer is observed, for which respective equilibrium constants can be determined. These equilibrium constants are in line with the equilibrium constant *K*_4_ for the formation of the hemiacetal **5** in the proposed mechanism of the Soai reaction (cf. Figure 3 and Table 2), which have been determined by comprehensive reaction networks analysis.

**Table 1:** Determination of equilibrium constants of the formation of hemiacetals from benzaldehyde and pyrimidyl-5-carbaldehydes by reaction with alcohols.

**Aldehyde**	**Alcohol**	**Hemiacetal**	**Yield**	***K***	***K*_**minor**_**	***K*_**major**_**
			**(%)**	**(M^**−1**^)**	**(M^**−1**^)**	**(M^**−1**^)**
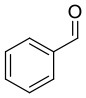	CD_3_OH	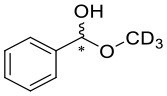	9	0.0040		
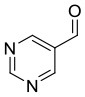 4_H_	CD_3_OH	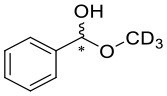	95	0.7733		
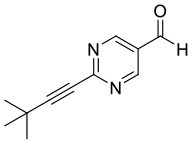 4	CD_3_OH	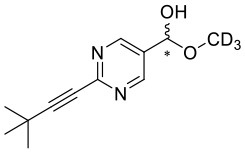	95	0.7722		
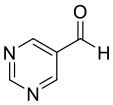 4_H_	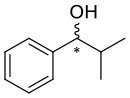	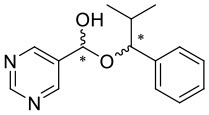	9	0.0544	0.0151	0.0393
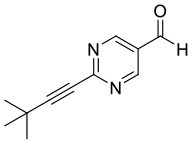 4	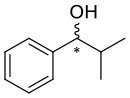	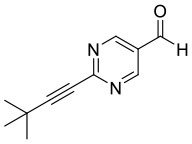	11	0.1195	0.0193	0.1002

*Quantification was performed by ^1^H NMR spectroscopy*.

**Table 2:** Kinetic data of the Soai-reaction of aldehyde **4** with *i*Pr_2_Zn forming alcohol **1** determined by comprehensive reaction networks analysis.

***n***	***k_***n***_***	***K_***n***_***	***k_**−*n***_***
1	1.5·10^2^ ± 7 M^−1^s^−1^		
2	7.0·10^2^ ± 32 M^−1^s^−1^	81 ± 4 M^−1^	8.6 ± 0.8 s^−1^
3	7.0·10^2^ ± 32 M^−1^s^−1^	162 ± 8 M^−1^	4.3 ± 0.4 s^−1^
4	1.7·10^−3^ ± 1.2·10^−4^ M^−1^s^−1^	0.136 ± 0.001 M^−1^	1.3·10^−2^ ± 1.0·10^−3^ s^−1^
5	63 ± 5 M^−2^s^−1^		
6	0.11 ± 0.01 s^−1^		
7	13.2 ± 0.2 M^−1^s^−1^		
8	0.23 ± 0.02 s^−1^		

This reaction network analysis was performed by using 26 differential equations describing the reaction kinetics of the reaction mechanism of the Soai reaction depicted in Figure 3 (see details in reference Trapp et al., 2020). These equations are implemented in a software program (Soai 7; Trapp, 2020). This program allows to calculate kinetic reaction profiles using an adaptive Runge–Kutta routine to solve the system of differential equations with the initial input concentrations of the enantiomers of the additive alcohol **1**, aldehyde **4**, and *i*Pr_2_Zn. The kinetic model allows to calculate kinetic reaction profiles of the conversion of the pyrimidine-5-carbaldehyde **4** into the reaction product **1** of the Soai reaction and the precise prediction of the non-linear amplification of the *ee* and the induction period in dependence on the *ee*.

**Figure 7:**
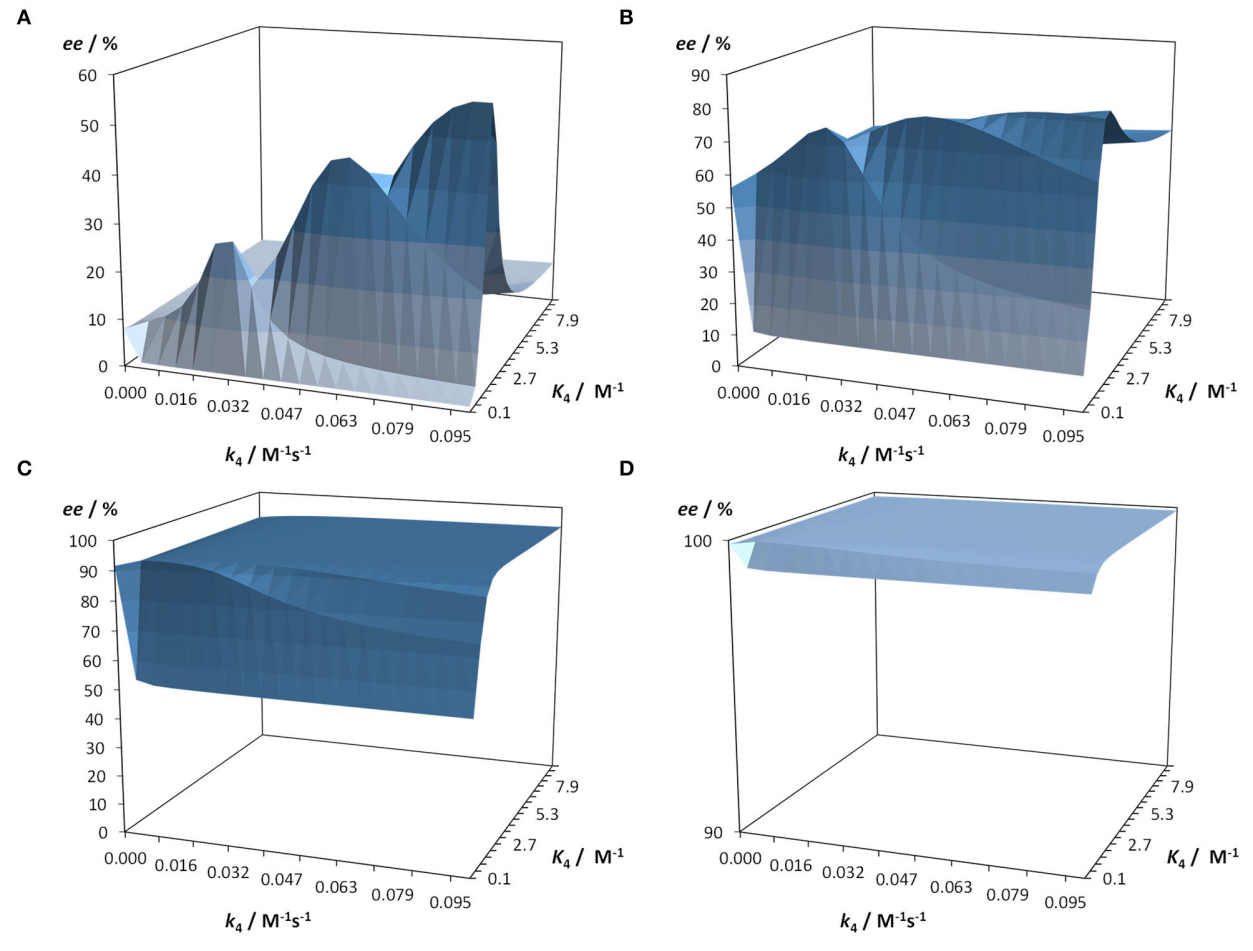
Prediction of the enantiomeric access in dependence of the reaction rate constant k4 and the equilibrium constant K4 of the hemiacetal formation. Starting conditions are 25 mM aldehyde and 40 mM iPr_2_Zn. Simulation time 1,200 s with a resolution of Δ*t* = 0.1 s. Calculations were performed with Soai 7. **(A)** Starting *ee* 1% [1.01 mM **(*R*)-1** and 0.99 mM **(*S*)-1**], **(B)** Starting *ee* 10% [1.1 mM **(*R*)-1** and 0.9 mM **(*S*)-1**], **(C)** Starting *ee* 50% [1.5 mM **(*R*)-1** and 0.5 mM **(*S*)-1**], and **(D)** Starting *ee* 99% [1.99 mM **(*R*)-1** and 0.01 mM **(*S*)-1**].

This kinetic model and software Soai 7 were used to investigate the influence of the reaction kinetics and equilibrium of the hemiacetal formation on the autocatalytic reaction and the enantiomeric excess *ee*. For this purpose the kinetic and thermodynamic parameters summarized in Table 2 were used and the parameters for the hemiacetal formation were varied in 20 steps each for *k*_4_ from 0.0001 to 0.1 M^−1^s^−1^ and for *K*_4_ from 0.05 to 10 M^−1^. Four scenarios were considered with increasing starting enantiomeric excess *ee*: *ee* 1% [1.01 mM **(*R*)-1** and 0.99 mM **(*S*)-1**] (cf. Figure 7A), *ee* 10% [1.1 mM **(*R*)-1** and 0.9 mM **(*S*)-1**] (cf. Figure 7B), *ee* 50% [1.5 mM **(*R*)-1** and 0.5 mM **(*S*)-1**] (cf. Figure 7C), and *ee* 99% [1.99 mM **(*R*)-1** and 0.01 mM **(*S*)-1**] (cf. Figure 7D). The predicted final high *ee'*s starting from already very high *ee*'s are not surprising. In this case the amplification (difference between final product *ee* and initial *ee*) is low. However, the simulation predicts that there are scenarios, which lead to a very amplification in a single step with proper *k*_4_ and *K*_4_ values. The result is, that a high stereodynamics with reaction rates *k*_4_ > 0.08 M^−1^s^−1^ paired with an equilibrium constant *K*_4_ in the range between 0.06 and 2.7 M^−1^ gives an immediate jump in the *ee* starting at 1% to an *ee* between 29 and 57%!

## Conclusions

By means of enantioselective dynamic HPLC (DHPLC) and ^1^H NMR studies the hemiacetal formation was investigated kinetically and thermodynamically. On the one hand, it could be shown that the formation process of the hemiacetal is endergonic and that there is a rapid conversion equilibrium between the hemiacetals. Simulations with the kinetic model of the Soai reaction under variation of the kinetics and thermodynamics of the hemiacetal formation allowed the prediction of the amplification of the enantiomeric excess depending on the addition of the alcohol as additive. The results show that in the underlying mechanism of the Soai reaction by the formation of transient stereodynamic hemiacetal catalysts the stereodynamics has an important influence on the resulting enantiomeric excess *ee*. It is remarkable that a high stereodynamics and equilibrium in favor of the alcohol and aldehyde compared to the hemiacetal leads to an enormous amplification of the enantiomeric excess *ee*. This leads to the conclusion that apparently inefficient processes lead to an optimal selection and amplification and thus to chirogenesis and homochirality. Furthermore, it can be concluded that maintaining homochirality is much more robust and tolerates wider ranges of kinetic and thermodynamic parameters.

## Data Availability Statement

The original contributions presented in the study are included in the article/supplementary materials, further inquiries can be directed to the corresponding author/s.

## Author Contributions

OT designed and performed the experiments and analysis, programmed the software application and wrote the manuscript.

## Conflict of Interest

The author declares that the research was conducted in the absence of any commercial or financial relationships that could be construed as a potential conflict of interest.

## References

[B1] Alberts A. H. Wynberg H. (1989). The role of the product in asymmetric carbon-carbon bond formation: stoichiometric and catalytic enantioselective autoinduction. J. Am. Chem. Soc. 111, 7265–7266. 10.1021/ja00200a059

[B2] Athavale S. V. Simon A. Houk K. N. Denmark S. E. (2020). Demystifying the asymmetry-amplifying, autocatalytic behaviour of the Soai reaction through structural, mechanistic and computational studies. Nat. Chem. 12, 412–423. 10.1038/s41557-020-0421-8 32203445 PMC7117993

[B3] Bissette A. J. Fletcher S. P. (2013). Mechanisms of Autocatalysis. Angew. Chem. Int. Ed. 52, 12800–12826. 10.1002/anie.201303822 24127341

[B4] Blackmond D. G. (2004). Asymmetric autocatalysis and its implications for the origin of homochirality. Proc. Natl. Acad. Sci. U.S.A. 101, 5732–5736. 10.1073/pnas.0308363101 15067112 PMC395976

[B5] Blackmond D. G. (2006). Mechanistic study of the Soai autocatalytic reaction informed by kinetic analysis. Tetrahedron: Asymmetry 17, 584–589. 10.1016/j.tetasy.2006.01.012

[B6] Blackmond D. G. (2010). Kinetic aspects of non-linear effects in asymmetric synthesis, catalysis, and autocatalysis. Tetrahedron: Asymmetry 21, 1630–1634. 10.1016/j.tetasy.2010.03.034

[B7] Blackmond D. G. (2011). The origin of biological homochirality. Phil. Trans. R. Soc. B 366, 2878–2884. 10.1098/rstb.2011.0130 21930578 PMC3158912

[B8] Blackmond D. G. (2020). Autocatalytic models for the origin of biological homochirality. Chem. Rev. 120, 4831–4847. 10.1021/acs.chemrev.9b00557 31797671

[B9] Blackmond D. G. McMillan C. R. Ramdeehul S. Schorm A. Brown J. M. (2001). Origins of asymmetric amplification in autocatalytic alkylzinc additions. J. Am. Chem. Soc. 123, 10103–10104. 10.1021/ja0165133 11592892

[B10] D'Acquarica I. Gasparrini F. Pierini M. Villani C. Zappia G. (2006). Dynamic HPLC on chiral stationary phases: a powerful tool for the investigation of stereomutation processes. J. Sep. Sci. 29, 1508–1516. 10.1002/jssc.200600129 16894796

[B11] Ercolani G. Schiaffino L. (2011). Putting the mechanism of the Soai reaction to the test: DFT study of the role of aldehyde and dialkylzinc structure. J. Org. Chem. 76, 2619–2626. 10.1021/jo102525t 21401089

[B12] Frank F. C. (1953). On spontaneous asymmetric synthesis. Biochim. Biophys. Acta 11, 459–463. 10.1016/0006-3002(53)90082-1 13105666

[B13] Gehring T. Quaranta M. Odell B. Blackmond D. G. Brown J. M. (2012). Observation of a transient intermediate in Soai's asymmetric autocatalysis: insights from 1H NMR turnover in real time. Angew. Chem. Int. Ed. 51, 9539–9542. 10.1002/anie.201203398 22936384

[B14] Girard C. Kagan H. B. (1998). Nonlinear effects in asymmetric synthesis and stereoselective reactions: ten years of investigation. Angew. Chem. Int. Ed. 37, 2922–2959. 10.1002/(SICI)1521-3773(19981116)37:21<2922::AID-ANIE2922>3.0.CO;2-1 29711141

[B15] Gridnev I. D. Vorobiev A. K. (2015). On the origin and structure of the recently observed acetal in the Soai reaction. Bull. Chem. Soc. Jpn. 88, 333–340. 10.1246/bcsj.20140341

[B16] Hawbaker N. A. Blackmond D. G. (2019). Energy threshold for chiral symmetry breaking in molecular self-replication. Nat. Chem. 11, 957–962. 10.1038/s41557-019-0321-y 31548669

[B17] Hegstrom R. A. (1984). Parity nonconservation and the origin of biological chirality: theoretical calculations. Origins life. 14, 405–411. 10.1007/BF00933684

[B18] Karunakaran S. C. Cafferty B. J. Weigert-Muñoz A. Schuster G. B. Hud N. V. (2019). Spontaneous symmetry breaking in the formation of supramolecular polymers: implications for the origin of biological homochirality. Angew. Chem. Int. Ed. 58, 1453–1457. 10.1002/anie.201812808 30537392

[B19] Kawasaki T. Suzuki K. Hakoda Y. Soai K. (2008). Achiral nucleobase cytosine acts as an origin of homochirality of biomolecules in conjunction with asymmetric autocatalysis. Angew. Chem. Int. Ed. 47, 496–499. 10.1002/anie.200703634 18058873

[B20] Kitamura M. Okada S. Suga S. Noyori R. (1989). Enantioselective addition of dialkylzincs to aldehydes promoted by chiral amino alcohols. Mechanism and Nonlinear Effect. J. Am. Chem. Soc. 111, 4028–4036. 10.1021/ja00193a040

[B21] Maier F. Trapp O. (2014). Selector-induced dynamic deracemization of a selectand-modified tropos BIPHEPO-ligand: application in the organocatalyzed asymmetric double-aldol-reaction. Angew. Chem. Int. Ed. 53, 8756–8760. 10.1002/anie.201402293 24965878

[B22] Micheau J.-C. Coudret C. Cruz J.-M. Buhse T. (2012). Amplification of enantiomeric excess, mirror-image symmetry breaking and kinetic proofreading in Soai reaction models with different oligomeric orders. Phys. Chem. Chem. Phys. 14, 13239–13248. 10.1039/c2cp42041d 22914796

[B23] Mikami K. Korenaga T. Ohkuma K. Noyori R. (2000). Asymmetric activation/deactivation of racemic Ru catalysts for highly enantioselective hydrogenation of ketonic substrates. Angew. Chem. Int. Ed. 39, 3707–3710. 10.1002/1521-3773(20001016)39:20<3707::AID-ANIE3707>3.0.CO;2-M 11091449

[B24] Satyanarayana T. Abraham S. Kagan H. B. (2009). Nonlinear effects in asymmetric catalysis. Angew. Chem. Int. Ed. 48, 456–494. 10.1002/anie.200705241 19115268

[B25] Scholtes J. F. Trapp O. (2019a). Design and synthesis of a stereodynamic catalyst with reversal of selectivity by enantioselective self-inhibition. Chirality 31, 1028–1042. 10.1002/chir.23132 31646689

[B26] Scholtes J. F. Trapp O. (2019b). Enantioselectivity induced by stereoselective interlocking: a novel core motif for tropos ligands. Chem. Eur. J. 25, 11707–11714. 10.1002/chem.201902017 31336015 PMC7522685

[B27] Scholtes J. F. Trapp O. (2019c). Inducing enantioselectivity in a dynamic catalyst by supramolecular interlocking. Angew. Chem. Int. Ed. 58, 6306–6310. 10.1002/anie.201901175 30786123

[B28] Scholtes J. F. Trapp O. (2019d). Supramolecular interlocked biphenyl ligands for enantioselective Ti-catalyzed alkylation of aromatic aldehydes. Organometallics 38, 3955–3960. 10.1021/acs.organomet.9b00262

[B29] Scholtes J. F. Trapp O. (2020). Stereoinduction and -Amplification in Stereodynamic Systems by Non-Covalent Interactions. Synlett 10.1055/a-1274-2777

[B30] Shibata T. Takahashi T. Konishi T. Soai K. (1997). Asymmetric self-replication of chiral 1,2-amino alcohols by highly enantioselective autoinductive reduction. Angew. Chem. Int. Ed. 36, 2458–2460. 10.1002/anie.199724581

[B31] Soai K. Kawasaki T. (2008). Asymmetric autocatalysis with amplification of chirality. Top. Curr. Chem. 284, 1–33. 10.1007/128_2007_138 16676331

[B32] Soai K. Shibata T. Morioka H. Choji K. (1995). Asymmetric autoctalysis and amplification of enantiomeric execss of a chiral molecule. Nature 378, 767–768. 10.1038/378767a0

[B33] Storch G. Deberle L. Menke J.-M. Rominger F. Trapp O. (2016a). A stereodynamic phosphoramidite ligand derived from 3,3'-functionalized ortho-biphenol and its rhodium(I) complex. Chirality 28, 744–748. 10.1002/chir.22655 27791317

[B34] Storch G. Pallmann S. Rominger F. Trapp O. (2016b). Stereodynamic tetrahydro-biisoindole “NU-BIPHEP(O)” s: functionalization, rotational barriers and non-covalent interactions. Beilstein J. Org. Chem. 12, 1453–1458. 10.3762/bjoc.12.141 27559397 PMC4979906

[B35] Storch G. Siebert M. Rominger F. Trapp O. (2015). 5,5'-diamino-BIPHEP ligands bearing small selector units for non-covalent binding of chiral analytes in solution. Chem. Commun. 51, 15665–15668. 10.1039/C5CC06306J 26361251

[B36] Storch G. Trapp O. (2015). Temperature controlled bidirectional enantioselectivity in a dynamic catalyst for asymmetric hydrogenation. Angew. Chem. Int. Ed. 54, 3580–3586. 10.1002/anie.201412098 25708097

[B37] Storch G. Trapp O. (2017). By-design enantioselective self-amplification based on non-covalent product-catalyst interactions. Nat. Chem. 9, 179–187. 10.1038/nchem.2638 28282051

[B38] Storch G. Trapp O. (2018). Supramolecular chirality transfer in a stereodynamic catalysts. Chirality 30, 1150–1160. 10.1002/chir.23007 30103282

[B39] Teichert J. S. Kruse F. M. Trapp O. (2019). Direct prebiotic pathway to DNA nucleosides. Angew. Chem. Int. Ed. 58, 9944–9947. 10.1002/anie.201903400 31131499

[B40] Trapp O. (2006a). Fast and precise access to enantiomerization rate constants in dynamic chromatography. Chirality 18, 489–497. 10.1002/chir.20276 16642493

[B41] Trapp O. (2006b). The unified equation for the evaluation of degenerated first order reactions in dynamic electrophoresis. Electrophoresis 27, 2999–3006. 10.1002/elps.200500907 16800031

[B42] Trapp O. (2006c). The unified equation for the evaluation of first order reactions in dynamic electrophoresis. Electrophoresis 27, 534–541. 10.1002/elps.200500708 16380957

[B43] Trapp O. (2006d). Unified Equation for access to rate constants of first-order reactions in dynamic and on-column reaction chromatography. Anal. Chem. 78, 189–198. 10.1021/ac051655r 16383327

[B44] Trapp O. (2013). Interconversion of stereochemically labile enantiomers (enantiomerization). Top. Curr. Chem. 341, 231–270. 10.1007/128_2013_453 23756848

[B45] Trapp O. (2020). Soai 7, Compatible With Microsoft Windows 7, 8 and 10. The compiled executable program can be obtained from the author upon request.

[B46] Trapp O. Bremer S. Weber S. K. (2009). accessing reaction rate constants in on-column reaction chromatography: an extended unified equation for reaction educts and products with different response factors. Anal. Bioanal. Chem. 395, 1673–1679. 10.1007/s00216-009-2993-4 19669676

[B47] Trapp O. Lamour S. Maier F. Siegle A. Zawatzky K. Straub B. F. (2020). *In situ* mass spectrometric and kinetic investigations of Soai's asymmetric autocatalysis. Chem. Eur. J. 26 (in press). 10.1002/chem.202003260 PMC7756584 32822103

[B48] Trapp O. Schoetz G. Schurig V. (2001). Determination of enantiomerization barriers by dynamic and stopped flow chromatographic methods. Chirality 13, 403–414. 10.1002/chir.1052 11466759

[B49] Tsogoeva S. B. (2010). When chiral product and catalyst are the same: discovery of asymmetric organoautocatalysis. Chem. Commun. 46, 7662–7669. 10.1039/c0cc02305a 20830352

[B50] Wolf C. (2008). Dynamic Stereochemistry of Chiral Compounds - Principles and Applications. Cambridge: RSC Publishing.

